# Manual of Clinical Diagnosis

**Published:** 1888-03

**Authors:** 


*Manual of Clinical Diagnosis.* By Dr. OTTO SEIFERT and Dr. FRIEDRICH MÜLLER. Translated by W. B. CANFIELD, M.D. Pp. 173. Edinburgh: YOUNG J. PENTLAND. 1887.

This is an exceedingly compact, thoroughly practical, and very comprehensive treatise on the whole subject of Clinical Diagnosis. It not only embraces all the regions, but it provides full descriptions of all the most recent methods of physical diagnosis. The book should become a favourite with English students; and will, no doubt, be appreciated by English teachers and practitioners as well.

